# Fibroblast Growth Factor 23 and Adverse Clinical Outcomes in Type 2 Diabetes: a Bitter-Sweet Symphony

**DOI:** 10.1007/s11892-020-01335-7

**Published:** 2020-08-28

**Authors:** Stanley M. H. Yeung, Stephan J. L. Bakker, Gozewijn D. Laverman, Martin H. De Borst

**Affiliations:** 1 Department of Internal Medicine, Division of Nephrology, University of Groningen, University Medical Center Groningen, P.O. Box 30.001, 9700 RB Groningen, the Netherlands; 2 Department of Internal Medicine/Nephrology, Ziekenhuisgroep Twente Hospital, Almelo and Hengelo, the Netherlands

**Keywords:** Mineral metabolism, Diabetes, Kidney disease, Cardiovascular disease

## Abstract

**Purpose of Review:**

Fibroblast growth factor 23 (FGF23) is a key phosphate-regulating hormone that has been associated with adverse outcomes in patients with chronic kidney disease (CKD). Emerging data suggest that FGF23 plays a specific role in type 2 diabetes, partly independent of kidney function. We aimed to summarize current literature on the associations between FGF23 and outcomes in patients with type 2 diabetes with or without CKD.

**Recent Findings:**

Several cohort studies have shown strong associations between plasma FGF23 and cardiovascular outcomes in diabetic CKD. Moreover, recent data suggest that FGF23 are elevated and may also be a risk factor for cardiovascular disease and mortality in type 2 diabetes patients without CKD, although the magnitude of the association is smaller than in CKD patients.

**Summary:**

Diabetes-related factors may influence plasma FGF23 levels, and a higher FGF23 levels seem to contribute to a higher cardiovascular and mortality risk in patients with type 2 diabetes. Although this risk may be relevant in diabetic individuals with preserved kidney function, it is strongly accentuated in diabetic nephropathy. Future studies should clarify if FGF23 is merely a disease severity marker or a contributor to adverse outcomes in type 2 diabetes and establish if antidiabetic medication can modify FGF23 levels.

## Introduction

Fibroblast growth factor 23 (FGF23) is a circulating hormone, predominantly produced by osteocytes, that regulates phosphate excretion by the kidneys and inhibits the synthesis of 1,25-dihydroxyvitamin-D_3_ [1^•^]. Initial studies found that deregulations in FGF23 play an important role in the development of bone and mineral disorders. Over 20 years ago, genetic mutations in the FGF23 gene were identified as the cause of autosomal dominant hypophosphatemic rickets (ADHR) [2]. Deregulated FGF23 also plays a role in the etiology of X-linked hypophosphatemic rickets (XLH), another bone disease where renal phosphate wasting plays a main role. Subsequently, novel therapies have been developed including the monoclonal antibody burosumab, which specifically targets FGF23. Clinical trials have now shown that burosumab restores phosphate metabolism, improves growth, and reduces pain in children with XLH [3]; it also seems to improve osteomalacia and skeletal complications in adults [4].

Since promoting renal phosphate excretion is among the main functions of FGF23, and since abnormalities in calcium-phosphate homeostasis are prominent in patients with chronic kidney disease (CKD), it seems plausible that FGF23 is involved in deregulated phosphate homeostasis in these patients. Indeed, many studies over the past years, including studies in patients with diabetic nephropathy, have shown that FGF23 levels are increased in CKD patients, most likely in response to a reduced renal capacity to excrete phosphate. In line, after successful kidney transplantation, FGF23 levels decline and transplanted patients may even develop transient hypophosphatemia [5, 6]. Moreover, deregulated FGF23 levels seem to have impact beyond bone disease, as high FGF23 levels have also consistently been associated with a higher risk of cardiovascular and all-cause mortality.

Patients with diabetic nephropathy may display a specific pattern of bone and mineral disturbances, including earlier and more severe increases in FGF23 levels. More recently, emerging data suggested that FGF23 levels may be specifically elevated in patients with type 2 diabetes and be associated with adverse outcomes irrespective of kidney function.

In this review, we will summarize the literature on potential factors driving FGF23 deregulation in diabetes. Subsequently, we address the known associations between FGF23 and outcomes in patients with type 2 diabetes who have either preserved or impaired kidney function.

## Elevated FGF23 Levels in Diabetes

Already in the 1980s, it was shown that patients with diabetic nephropathy have a lower bone mass than non-diabetic CKD patients and that circulating parathyroid hormone (PTH) levels are relatively low [7, 8]. Also, it has been long known that patients with type 2 diabetes are more prone to fractures and have a higher bone mineral density and an abnormal bone formation rate compared with non-diabetic patients [9–12]. To expand on this, it seems that diabetic patients who have microvascular complications have more severe bone abnormalities compared with patients without microvascular complications, suggesting that microangiopathy might cause bone abnormalities in patients with diabetes [13^•^]. Furthermore, diabetes may also interfere with bone and mineral metabolism. Recent studies have provided a more detailed overview of the spectrum of bone and mineral abnormalities in diabetic nephropathy, including data on FGF23 (Table 1). Initial studies were already suggestive of deregulated FGF23 levels in diabetes, compared with non-diabetic controls, but the limited sample size and heterogeneity of these studies could have confounded the results [17, 18]. From a large analysis of the Chronic Renal Insufficiency Cohort (CRIC) study, Wahl et al. concluded that the presence of co-existing diabetes was independently associated with higher levels of serum phosphate, PTH, and FGF23 [14••]. Moreover, FGF23 excess was more prevalent earlier in the course of CKD among those with diabetes versus those without diabetes [14••]. The higher FGF23 levels in diabetic nephropathy may at least partly explain the previously observed association between diabetes and lower 1,25(OH)_2_-vitamin D levels [19]. Túñon et al. found similar FGF23 levels in diabetic vs. non-diabetic patients; this could be explained by the fact that these patients had better kidney function as compared with the CRIC study [15•]. On the other hand, a smaller cohort of prediabetic individuals with normal kidney function displayed significantly higher intact FGF23 levels than obese controls with normal glucose tolerance [16]. In a previous study, we assessed FGF23 in a general population cohort, which also included type 2 diabetes patients [20••]. Here, we specifically analyzed type 2 diabetes patients with an estimated glomerular filtration rate (eGFR) > 60 ml/min/1.73m^2^ and matched these patients with individuals without diabetes using propensity score matching analysis (Table 2). After matching, FGF23 levels remained higher in patients with type 2 diabetes than in patients without diabetes (75.6 [IQR 61.3–91.8] vs. 70.8 [IQR 58.0–85.8] RU/mL, respectively, *P* < 0.001). While the conditions that determine FGF23 levels in patients with diabetes and preserved kidney function are not entirely clear, there seems to be interaction between diabetes and kidney function, with the highest FGF23 levels present in patients with both diabetes and impaired kidney function.

**Table 1:** Overview of studies comparing FGF23 levels in (pre-)diabetes patient with controls

Author	N	Age (years)	eGFR (ml/min/1.73m^2^)	FGF23 assay	FGF23 level
Wahl et al. [14••]					
Type 2 diabetes patients	1820	59.5 ± 9.8	40.7 ± 12.8	C-terminal	172.4 (114.3–277.2) RU/mL*
Non-diabetic controls	1936	57.0 ± 11.9	44.7 ± 13.8	C-terminal	121.9 (84.0–198.8) RU/mL
Tuñón et al. [15•]					
Type 2 diabetes patients	173	62.8	73.8 ± 20.8	C-terminal	72.2 (56.7–104.9) RU/mL
Non-diabetic controls	531	61.0	76.5 ± 18.0	C-terminal	69.9 (54.4–93.7) RU/mL
Gateva et al. [16]					
Prediabetes patients	39	50.3 ± 11.5	124.5 ± 39.8	Intact	10.4 ± 10.7 pg/mL*
Non-diabetic obese	41	50.6 ± 9.7	125.2 ± 35.6	Intact	5.8 ± 7.3 pg/mL

*P < 0.05 vs controlsAbbreviations: *eGFR* estimated glomerular filtration rate, *cFGF23* C-terminal fibroblast growth factor-23

**Table 2:** Patient characteristics before and after propensity score matching between diabetes patients and non-diabetic controls in the PREVEND study

	Type 2 DM (*n* = 288)	Non-diabetic controls (*n* = 5352)	P^*^	SMD^†^	Controls after matching (*n* = 288)	SMD
C-terminal FGF23, (RU/mL)	75.6 (61.3–91.8)	67.9 (55.9–85.0)	**< 0.001**	0.12	70.8 (58.0–85.8)^§^	0.16
Age, (yrs)^**‡**^	62 ± 10	52 ± 11	**< 0.001**	0.95	61.7 ± 10.8	0.001
Men, n (%)^**‡**^	144 (50)	2462 (46)	0.10	0.08	150 (52)	0.04
BMI, (kg/m^2^) ^**‡**^	29.4 (26.4–32.5)	25.8 (23.5–28.7)	**< 0.001**	0.79	29.0 (26.3–32.9)	0.02
Systolic blood pressure, (mmHg)	137 ± 20	124 ± 18	**< 0.001**	0.68	138 ± 21	0.03
Diastolic blood pressure, (mmHg)	76 ± 9	73 ± 9	**< 0.001**	0.38	77 ± 9	0.04
eGFR (CKD-EPI), (mL/min/1.73m^2^)^**‡**^	85.4 (74.3–98.2)	93.8 (80.7–108.4)	**< 0.001**	0.44	85.6 (74.8–97.0)	0.04
Plasma phosphate, (mmol/L)^**‡**^	1.02 ± 0.2	1.02 ± 0.3	0.62	0.03	1.04 ± 0.5	0.07
Plasma PTH, (pmol/L)	5.0 (4.2–5.9)	4.9 (4.1–5.8)	0.13	0.08	5.2 (4.1–6.1)	0.03
Plasma vitamin D3, (nmol/L)^**‡**^	45.5 (33.4–62.5)	53.6 (38.3–72.3)	**< 0.001**	0.33	47.4 (34.3–63.6)	0.04
Urinary P excretion, (mmol/24 h)^**‡**^	15 (10–21)	15 (10–22)	0.23	0.07	14 (10–22)	0.03

Propensity score-based matching (1:1) with all covariates displayed in the table

*P value represents differences between the groups before matching assessed by student’s *t* test or Mann–Whitney U test for nominal and non-normally distributed data, respectively. Chi-squared test was used for categorical variables

**†** The standardized mean difference (SMD) compares the difference in the mean in units of the standard deviation of both groups. SMD <0.1 after matching represents a negligible difference of the covariate between the groups

§ *P < 0.001* between the groups after propensity score matching using Mann–Whitney U test

Abbreviations: *SMD* standardized mean difference, *FGF23* fibroblast growth factor 23; *eGFR* estimated glomerular filtration rate, *HS CRP* high sensitive C-reactive protein, *PTH* parathyroid hormone, vitamin D3, 25-OH, 25-hydroxycholecalciferol, *P* phosphate, *ACR* albumin-to-creatinine ratio

## Potential Pathways Driving FGF23 in Diabetes

**Fig. 1:**
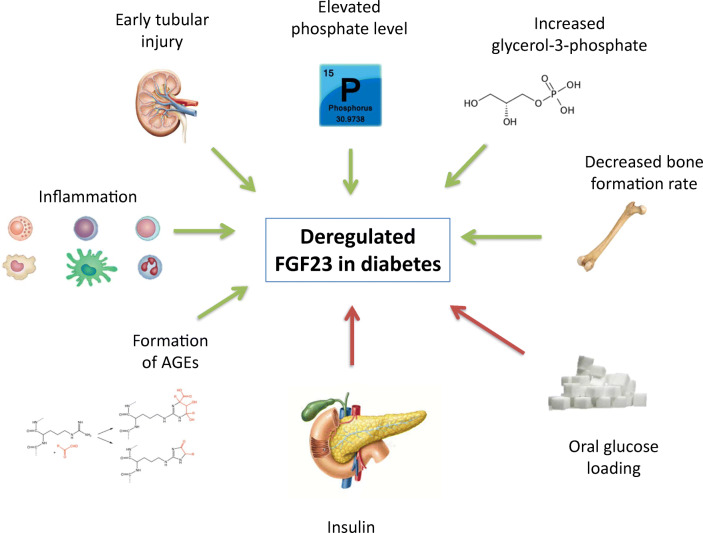
Overview of factors that may contribute to deregulation of FGF23 in diabetes. Factors that may increase FGF23 levels are indicated with green arrows, while factors that may reduce FGF23 levels are indicated with red arrows

Several factors may contribute to deregulated FGF23 in patients with diabetes (Fig. 1). *First*, patients with diabetic nephropathy seem to have higher serum phosphate levels than matched controls without diabetes [14, 17]. Consequently, FGF23 levels may be elevated in order to keep phosphate balance by stimulating renal phosphate excretion. *Second*, a recent series of elegant experiments identified glycerol-3-phosphate (G3P), a metabolite involved in glycolysis, as a major FGF23 regulator in the setting of acute kidney injury [21••]. Diabetes is characterized by impaired mitochondrial functioning and dysregulated G3P metabolism [22, 23]. Thus, although the initial discovery linking G3P with FGF23 production was in acute kidney injury, this concept also provides an hypothetical link between dysregulated G3P metabolism and FGF23 levels in patients with diabetes. *Third*, decreased bone formation rates as observed in patients with diabetes could be a stimulus for FGF23 secretion [24]. *Fourth*, oral glucose loading seems to lower FGF23 levels independent of insulin in patients with an impaired glucose tolerance; whether this is a direct effect of glucose on bone remains to be established [25]. *Fifth*, patients with diabetes are more prone to develop early tubular injury, prior to a measurable decrease in kidney function or albuminuria [26]. Since FGF23 target tubular epithelial cells to promote phosphaturia, early tubular dysfunction could at least partly contribute to higher FGF23 levels in diabetes [14••]. *Sixth*, high levels of glucose could lead to formation of advanced glycation end products (AGEs) [27]. It has been suggested that AGEs could induce a higher FGF23 levels [28]. *Seventh*, inflammation is a major trigger of FGF23 production [29], and most patients with type 2 diabetes, especially those with obesity, are in a pro-inflammatory state [30]. *Eighth*, a recent study found that insulin and insulin-like growth factor are strong suppressors of FGF23 in animals and humans [31]. This may explain high FGF23 levels in type 1 diabetes, whereas in type 2 diabetes hyperinsulinaemia may be expected to lead to lower FGF23 levels. Possibly, in type 2 diabetes, the aforementioned pro-inflammatory state may overrule the suppressive effect of hyperinsulinaemia, ultimately leading to higher FGF23 levels overall.

Elevated FGF23 levels may, in turn, also influence glucose homeostasis. In mice, knockout of the FGF23 gene results in a specific phenotype characterized by, among others, hypoglycemia and increased peripheral insulin sensitivity [1, 32]. Less is known on the effects of FGF23 on glucose metabolism in humans. A number of smaller studies suggest an inverse correlation between FGF23 levels and insulin sensitivity [33–36], which would be in line with preclinical data. FGF23 is positively associated with resistin, an adipokine, and regulator of insulin resistance, irrespective of kidney function [36, 37]; it should be further addressed whether this relationship is key to FGF23-induced insulin resistance. Even though the underlying mechanisms are not entirely clear, emerging data indicate that a higher FGF23 level may influence clinical outcomes.

## FGF23 in Chronic Kidney Disease

Although the main focus of this review is on FGF23 in diabetes, most data on the association between FGF23 and clinical outcomes are available in patients with CKD. A landmark study published in 2008 showed that FGF23 levels were independently associated with a higher mortality risk in end-stage kidney disease patients requiring hemodialysis [38]. A wide range of subsequent cohort studies consistently showed that worsening kidney function is accompanied by gradually higher FGF23 levels [39] and that an elevated circulating FGF23 level is associated with a higher mortality risk across stages of CKD [38, 40••] and in kidney transplant recipients [41–43]. Particularly, CKD patients with a rapidly increasing FGF23 level seem to have a higher mortality risk [44]. At least part of the excess mortality seems to be attributable to cardiovascular disease [42] and, more specifically, FGF23 has been linked with a higher risk of (progressive) heart failure [45, 46]. FGF23 has also been associated with a higher risk of kidney function decline in patients with CKD [40••] and with an increased risk of developing CKD in the general population [47••]. Although it remains unclear whether FGF23 or its receptor may be causal factors that promote kidney damage or impair renoprotective therapy [48, 49], emerging data indicate that FGF23 can have detrimental off-target effects on the heart. A meta-analysis observed that in different CKD stages FGF23 is indeed associated with cardiovascular outcomes and mortality, but it was also associated with non-cardiovascular outcomes. This indicates that it may not only have an effect on cardiovascular system, but also on other organ systems [50•].

## FGF23 and Outcomes in Diabetes

As outlined above, in patients with CKD, there seems to be a rather consistent association between FGF23 levels and adverse outcomes across CKD stages, while absolute FGF23 levels are much higher with more advanced CKD stages. Similarly, these associations appear independent of the primary kidney disease. Several studies have demonstrated that in patients with type 2 diabetes, a higher circulating FGF23 level is associated with an increased risk of all-cause mortality and cardiovascular mortality (Table 3). We also analyzed cardiovascular events as a separate outcome and found that a higher FGF23 level is also associated with a higher risk of cardiovascular events (cardiovascular mortality, unstable angina pectoris, myocardial infarction, transient ischemic attack, cerebrovascular diseases (cerebral infarction or hemorrhage), or heart failure) [20••].

**Table 3:** Studies assessing the relationship of FGF23 with outcomes in patients with type 2 diabetes

Author	N	Follow-up (years)	Age (years)	eGFR (ml/min/1.73 m^2^)	FGF23 (RU/mL)	Outcome: hazard ratio (95% CI)^#^
Silva et al. [51]	107	2.8 ± 0.7	57.2 ± 7.1	52.89 ± 20.15	135.0 ± 135.2	CV mortality: 2.05 (1.01–8.25)
Titan et al. [52]	55	2.6 ± 0.8	58.4 ± 10.0	53.0 ± 20.6	92.0 ± 42.9	Composite endpoint^1^: 1.09 (1.01–1.16)
Tuñón et al. [15•]	173	2.15 ± 0.99	62.8	73.75 ± 20.84	72.2 (56.7–104.9)	Composite endpoint^2^: 1.27 (1.13–1.43)
Yeung et al. [20••]	310	5.8 (3.3–6.5)	61.5 ± 8.7	88.5 ± 14.8	84.2 (67.0–117.6)	All-cause mortality: 2.55 (1.58–4.10) MACE: 1.68 (1.08–2.61)
Frimodt et al. [53]	200	6.1 (5.9–6.6)	59.9 ± 9	91.1 ± 18.3	71 (52–108)	All-cause mortality: 1.57 (1.11–2.18)
Chan et al. [54]	513	6.6 (5.8–7.5)	55.0 (49.0–62.0)	91.3 (76.4–111.3)	112.4 (79.0–165.8)	All-cause mortality: 1.74 (1.44–2.09)

^1^Composite endpoint of all-cause mortality, doubling of serum creatinine or requirement for dialysis

^2^Composite endpoint of acute ischemic events (acute coronary syndrome, stroke or transient ischemic attack), heart failure or death

^#^Adjusted for potential confounders

Abbreviations: *eGFR* estimated glomerular filtration rate, *FGF23* fibroblast growth factor 23, *CI* confidence interval, *CV* cardiovascular, *MACE* major adverse cardiac event

Interestingly, the association between FGF23 and adverse outcomes in diabetes seems to extend beyond patients with impaired kidney function, as the majority of patients in some of the studies summarized in Table 3 had an eGFR > 60 mL/min/1.73 m^2^, and in two studies, the average eGFR was even > 90 mL/min/1.73 m^2^ [53, 54]. This is in line with a specific role for FGF23 in patients with type 2 diabetes, even in patients with preserved or mildly impaired kidney function.

## Potential Mechanisms Linking High FGF23 Levels with Outcomes

FGF23 was observed to be associated with different outcomes, which has led to studies investigating the consequences of excess FGF23 levels. The primary targets of FGF23 in the kidney are NaPi2a/c sodium/phosphate co-transporters, via signaling through the FGF receptor 1 (FGFR1) in conjunction with the canonical co-receptor α-klotho. Given its prominent role as a regulator of phosphate homeostasis, FGF23 may contribute to cardiovascular disease through deregulation of phosphate balance, promoting vascular calcification through increased phosphate deposition in the vascular wall. Some [55], but not all [56, 57] studies have linked a higher FGF23 level with vascular calcification. Furthermore, phosphaturia induced by FGF23 may lead to interstitial inflammation, fibrosis, and tubular damage [58]. Although vascular calcification is a prominent feature of cardiovascular disease in diabetes, particularly with co-existing CKD, it does not seem to be the only pathway linking FGF23 with adverse outcomes.

In addition to the regulation of phosphate reabsorption, a higher FGF23 level may also increase renal sodium uptake, leading to volume expansion and hypertension [59–61]. Although these data are not entirely consistent, it may at least partly explain the role of FGF23 in cardiovascular disease as observed in epidemiological studies. Additionally, so-called “off-target” FGF23 signaling routes in different cell types have been identified; for the cardiovascular effects of FGF23 the most relevant off-target signaling pathway seems to be through FGFR4. FGF23 can bind and activate FGFR4 in the heart, independently of α-klotho [62], promoting left ventricular hypertrophy [63, 64]. Importantly, from preclinical studies, it seems that pharmacological interference with a specific FGFR4 inhibitor might protect from CKD- and age-related left ventricular hypertrophy [65], opening up novel avenues for intervention aiming to lower the massively elevated cardiovascular risk in CKD. This is particularly relevant since more conventional efforts to lower FGF23, using a phosphate-restricted diet and phosphate binders, have been barely successful [66].

Also worth mentioning is that FGF23 might have an interaction with asymmetric dimethylarginine (ADMA), as it is suggested that both can interfere with the nitric oxide system leading to endothelial dysfunction and atherosclerosis which is associated with CKD progression [67]. High FGF23 levels may also influence α-Klotho [68] and as animal studies have shown that a reduction of renal Klotho may result in kidney damage through pro-fibrotic signaling pathways, including transforming growth factor β1 and Wnt/β-catenin signaling [69, 70].

## FGF23-Reducing Strategies

In the literature, many FGF23-reducing strategies have been extensively studied. Initial studies focused on strategies that lower serum phosphate levels by restricting dietary phosphate intake [71, 72] and/or using phosphate binders [73]. However, discrepant results were observed, as some studies reported no effect on FGF levels of dietary phosphate restriction [74, 75] and/or phosphate binders, alone or in combination with nicotinamide [76, 77]. Therefore, the effectiveness of these strategies remain uncertain. Other approaches include the use of monoclonal antibodies against FGF23 like burosumab. However, in a preclinical study FGF23 monoclonal antibodies applied in a rat model of CKD-mineral and bone disorder led to normalization of bone and mineral markers but increased aortic calcification and mortality, probably due to an incapacity to excrete phosphate, which was already impaired due to the CKD background [78]. Based on these data, it seems unlikely that isolated anti-FGF23 therapy is beneficial in the context of impaired kidney function. The same results were found using a pan-FGFR inhibitor showing that FGF23 is an important phosphate-regulating hormone [79]. Some studies have found that calcimimetics are a viable option to reduce FGF23 levels [80, 81]; however data have so far been limited to patients with end-stage kidney disease. Although theoretically cross-talk between the renin-angiotensin system and FGF23 may lead to changes in FGF23 levels in response to angiotensin-converting enzyme inhibitor (ACEi) or angiotensin receptor blocker (ARB) treatment, so far no studies have demonstrated this convincingly to our knowledge [82]. Interventions aiming to optimize renin-angiogensin-aldostero system (RAAS)-blockade efficacy, such as low-sodium diet or the addition of a thiazide diuretic, may not affect FGF23 levels in diabetic nephropathy [61].

The aforementioned strategies were mostly performed on CKD patients, and thus these strategies have yet to be studied specifically in patients with type 2 diabetes. It is of interest whether antidiabetic medication may influence FGF23 levels in diabetic nephropathy. We recently demonstrated that treatment of diabetic nephropathy patients with the sodium-glucose cotransporter inhibitor (SGLT2i) dapaglifozin led to small but significant increases in serum phosphate, plasma PTH, and FGF23, independent of concomitant changes in eGFR or 24-h albumin excretion [83••]. A potential mechanism may be that since phosphate and glucose transporters use the same sodium gradient, these transporters may limit each other [84]. Because SGLT2 inhibitors prevent the cotransport and reabsorption of sodium and glucose, the sodium gradient is preserved for the sodium-dependent phosphate transport proteins NaPi-2a and NaPi-2c, stimulating tubular phosphate reabsorption [85]. Despite these small effects on mineral metabolism, SGLT2 inhibitors have shown clinically relevant cardiovascular and renal protective effects in diabetic nephropathy, without convincing adverse effects on the bone [85•]. As mentioned before, insulin seems capable to reduce FGF23 levels in human and mice [31]; the clinical value of this observation should be further addressed. Overall, future studies should evaluate the value of antidiabetic medication in reducing FGF23, to further clarify the role of FGF23 in patients with diabetes.

## Conclusions

The current literature indicates that patients with type 2 diabetes, and particularly those with impaired kidney function, generally have an increased FGF23 level compared with individuals without diabetes. This is most likely the consequence of a complex interplay of several deregulated pathways that co-occur in diabetic nephropathy. Importantly, although the underlying pathways have not been fully clarified, a higher FGF23 level has been strongly and consistently linked with a higher risk of (cardiovascular) morbidity and mortality. Interestingly, these associations have been observed in patients with reduced and preserved kidney function, highlighting the prominence of FGF23 as a risk factor in patients with diabetes.

The next question is whether FGF23 plays a causal role in these adverse outcomes or whether it is rather an indicator of an underlying dismal process such as inflammation. In order to address this question in detail, interventions specifically targeting FGF23 need to be studied in diabetic animals and patients with preserved kidney function as many possible therapeutic interventions was applied in the context of impaired kidney function. It may be interesting to study whether such therapy could benefit individuals with diabetes and preserved kidney function, possibly in combination with reduced phosphate intake. Future studies are clearly needed to further advance this field and better understand the deregulations in phosphate metabolism, including FGF23, in patients with diabetes.
